# An Evaluation of Alternatives for Providing Care to Veterans

**DOI:** 10.3390/healthcare6030092

**Published:** 2018-08-02

**Authors:** Lawrence V. Fulton, Matthew S. Brooks

**Affiliations:** Department of Health Administration, Texas State University, HPB 250, 601 University Drive, San Marcos, TX 78666, USA; mbrooks@txstate.edu

**Keywords:** health policy, VHA, VA, CMS TRICARE, scandal

## Abstract

In 2014, a whistleblower reported that many U.S. veterans died while waiting for care at the Phoenix VHA. Problems with veteran’s care through 2018 reveal ongoing and systematic problem. In March 2018, the VA Inspector General identified critical deficiencies at the Washington, DC VA Medical Center including failures to track patient safety events accurately, ineffective sterile processing and more than 10 thousand open or pending prosthetic/sensory aid consults. The VHA clearly has problems with access and quality in a budget-constrained environment. In this policy analysis, four separate interventions that address the gap between the magnitude as well as the use of the VHA’s fixed budget versus access and cost expectations are explored. These policy interventions include maintaining the status quo, returning to a “VHA-only” option, transitioning to a CMS central payer system and consolidating care under the DoD TRICARE insurance plans. An objective evaluation suggests that extending TRICARE to veterans during the phasing out the VHA’s care responsibilities, while politically unpalatable, would likely provide the best of four possible solutions under various criterion weighting schemes. A central payer solution under the CMS would also be a viable consideration. Results suggest that TRICARE patient perceptions of quality are superior to VHA and non-VHA/non-DoD, that access provided by the TRICARE program is ranked second in terms of venue acceptance only to the CMS solution set based on primary provider acceptance and that the cost per beneficiary of a TRICARE solution ($6.5 K/beneficiary) is far better than a VHA-only solution ($14.0 K/beneficiary), the CMS central payer solution ($12.2 K/beneficiary), or the status quo (between $12.2 K and $14.0 K/beneficiary). The intent of this paper is to provoke thoughtful consideration of solutions for providing access to high-quality healthcare for veterans within or outside of the VHA.

## 1. Introduction

The United States Veteran’s Health Administration (VHA) is the “largest integrated healthcare system, providing care at 1240 facilities, including 170 medical centers and 1061 outpatient sites of care of varying complexity (VHA outpatient clinics), serving 9 million enrolled veterans each year” [1]. For FY2019, the VHA will maintain more than 315,000 full-time equivalents and a budget (both mandatory and discretionary with stepped down administrative costs) estimated to be nearly $98 billion [2]. The organization’s mission is to “Honor America’s Veterans by providing exceptional health care that improves their health and well-being” [3]. Unfortunately, the VHA’s recent and historical problems with mismanagement, reporting falsification and preventable deaths have made it “exceptional” in unfortunate ways. This problem has been characterized as “system-wide” by Concerned Veterans of America [4]. These recent problems provide the United States an opportunity to evaluate alternatives that provide veterans cost-effective and quality-centered health services. This policy analysis paper identifies the VHA system problems and ongoing policy interventions, evaluates possible additional policy interventions by drawing from other systems external and internal to the US, analyzes these possible interventions against developed criterion and leverages utility matrices to evaluate them.

### 1.1. Problem Background

In 2014, a whistleblower reported that “at least 40” U.S. veterans died while waiting for care at the Phoenix VHA. More than 1400 of these veterans were placed on a “secret” waiting list in an apparent effort to hide the actual treatment delays [5]. The VA Office of the Inspector General’s (IG’s) investigation confirmed the existence of an “off-book” waiting list and further identified 28 cases of clinically significant delays with 6 associated deaths. By August of 2014, the VA IG was investigating 93 VHA facilities for manipulating wait times but had confirmed manipulations of wait time were prevalent. One of the conclusions of the IG was that emphasis on goals (linked to financial incentives) provided a “misleading portrayal” of veterans’ access and that the reported outcomes were both inaccurate and unsupported [6]. The demand from high-need returning veterans likely outstripped the supply and the VHA handled it poorly [7].

Similar misconduct occurred in the United Kingdom. Between January 2005 and March 2009, Britain’s National Health Service (NHS) experienced a scandal, which was discovered when mortality rates were increasing [8]. The UK government was incentivizing frugality by offering autonomy and a share of savings for those facilities attaining “Foundation” status [9]. The focus on performance targets was associated with an unexpected increase in deaths during this period (estimated to be as many as 1200). In another example from 2001, the National Audit Office identified nine trusts that were inappropriately manipulating waiting lists, identical to the problem of the VHA. Some of the problems identified in the report include altering of patient records, deletions from waiting lists and non-reporting of patients waiting 18 months or more. The report found that some managers simply sought to meet performance benchmarks [10].

In both government-run and government-funded organizations (the VHA and NHS), management’s focus appeared to be on performance targets rather than patients and incentives for fraud existed. In the case of the VHA, the problems continue today. A recent report from the VA IG confirmed that the VHA’s Eastern Colorado Health Care System was using “off-book” patient scheduling which under-reported treatment delays [11]. Waiting time manipulation since 2014 has occurred in at least seven states [12]. In 2016, the Phoenix VHA again became the subject of investigation, as VA IG inspectors identified 215 deceased patients who were awaiting specialist consultations on the date of death. Staff were still not managing or scheduling consults according to VHA policy [13].

### 1.2. Problem and Significance

The problem for the VHA and under consideration here is the identification of possible policy solutions that improve on status quo performance and address the gap between the magnitude as well as the use of the VHA’s annual and performance expectations for quality and access [8]. The significance of this problem may be measured in mortality and morbidity. The true scope of the problem is unknown, as metrics are only available for those who successfully accessed the system. Those veterans discouraged with access issues may not seek care and are of unknown quantity.

Like the NHS, the VHA operates with an annual budget and has certain benchmarks for quality and access. This budget versus benchmark relationship may be irreconcilable at the current operating efficiency level. Policy efforts have attempted to fill the budget versus benchmark gap by increasing funding and access; however, the success of these interventions has been mixed. The purpose of this policy analysis is to assess current and future policy interventions that might address the requirement to provide veterans access to high-quality, cost-effective healthcare.

### 1.3. Previous Policy Interventions

As a direct response to the 2014 VHA scandal, President Obama signed the Veterans Access, Choice and Accountability Act of 2014 (PL 113-146). This law approved $10 billion for the Veterans Choice plan, authorizing civilian care opportunities to veterans who live more than 40 miles from a VA facility or who wait for more than 30 days for healthcare. Further, it offered another $5 billion to the VHA for personnel and facilities, prevented bonus payments from being tied to wait-time goals, implemented additional oversight of facilities and provided the VA Secretary expanded authority to fire poorly performing managers. Initial funding for Veterans Choice ended on 31 May 2018 [14]. A 2017 NPR investigation, however, revealed that the VA hired no more providers than it would have without the funding, that the new hires were not sent to the hospitals with the longest waiting times and that the facilities which received the new hires were not more likely to see improved waiting times [15].

On 6 June 2018, President Trump signed the VA Maintaining Systems and Strengthening Integrated Outside Networks Act (VA Mission Act of 2018). This act (among other things) provided a community health program for veterans and extended Veterans Choice coverage until the Mission Act is implemented. Under the new law, veterans are entitled access to community care if the VHA does not offer the care or services required, it does not operate a full-service medical facility in the veteran’s state, the veteran lives more than 40 miles from a VHA facility, the VHA is not able to meet the designated access standards it establishes, or the veteran and clinician agree that community care would be in the veteran’s best interest [16]. The Congressional Budget Office (CBO) estimates the VA Mission Act will cost $55.2 billion over 5 years with $5.2 billion extending the Veterans Choice program, $4.5 billion extending certain Medicaid-eligible veteran pension benefits and $46.5 billion for the community healthcare program [17].For FY2019, $14.2 billion is provided for community care [2].

Coupled with this $14.2 billion is the VHA FY2019 requested budget of $76.5 billion for medical care, $1.2 billion for an electronic health record (EHR) system, $0.7 billion for research and $1.5 billion for construction for a total of $79.9 billion [2].Assigning 80% of the requested $4.2 billion for informational technology, $0.4 for general administration and $0.2 for IG functions to the VHA (due to its sheer size relative to the other organizations within the VA) results in an adjusted VHA estimated budget of $83.8 billion. While the organization reports on its website that there are 9 million enrolled veterans, its budget request is for 7.0 million treated veterans [2]. NOTE: A veteran “enrolled” in the VHA may never use the system, as he or she may have secondary insurance such as TRICARE (e.g., military retirees) or through other employment, so the “treated veterans” is likely the better estimate of actual beneficiaries. This dual eligibility provides some complication when accounting for the number of true beneficiary users, particularly if users split care between systems. With the $14.2 billion for community care, the total cost for veteran care becomes an estimated $98 billion or $14.0 K per veteran per year. Understanding this baseline becomes important when considering additional policy alternatives.

### 1.4. Why Are Policies Shifting towards External Care for Veterans?

Veterans Choice and the VA Mission Act pave an avenue to civilian care for veterans, which is a shift in policy. The question becomes “why?” The VA indicates that “there is no effort underway to privatize [6] VA and to suggest otherwise is completely false and a red herring designed to distract and avoid honest debate on the real issues surrounding Veterans’ health care” [18]. In this statement, the VA points to increases in budget, employees and facilities over two decades. President Trump has indicated a shift in policy towards joint community and VHA care, by stating, “We’re allowing our veterans to get access to the best medical care available, whether it is at the VA or at a private provider” [19]. Further, the VA Mission Act also established a commission charged with reviewing the VA infrastructure assets for closure (i.e., the 1240 facilities), a commission that will not have any VA representation [20]. It is possible that there will be a blended VA system for quite some time; however, it is fathomable that the incorporation of external healthcare providers may be logical incrementalism [21], a slow move away from the VHA’s NHS-like structure in favor of a single payer system for veterans. From a broader perspective, it is also possible that the VHA may see transformational change through additional policy.

### 1.5. Some Major Stakeholders

Some of the major actors in the veterans’ healthcare crisis problem are the veterans themselves, veterans’ groups, the public, the VHA, the Congressional Democrats, the Congressional Republicans, the Executive Branch and the media. Each of these stakeholders has evidence-based positions on policy.

#### 1.5.1. Veterans, Veterans’ Groups and the Public

A somewhat-dated poll (2015) indicates that 64% of veterans oppose “privatization” of the VHA system, although that definition is not well defined [22]. Veterans’ groups such as the Concerned Veterans for America have emerged as powerful players in this administration [23]. The public itself is influential and there is highest level of grass-roots support for increased spending on veterans in two decades [24].

#### 1.5.2. The VHA

The VHA opposes any policy that results in further dilution of organic management and treatment [18]. But the VHA may have little power in the process as evidence by the fact that the VA Mission Act excludes them from even having a representative on the committee that will evaluate infrastructure for elimination [20].

#### 1.5.3. Congress and the President

Democrats and Republicans demonstrated significant bi-partisanship when passing the VA Mission Act, with the Senate voting 92% in favor and the House voting 81% in favor. Both parties officially oppose “privatization,” an ill-defined buzzword which may trigger emotional responses in constituencies [25]. Republicans are characteristically more supportive of small government [26] relative to Democrats, so one might assume their support for a smaller VHA. As a former business leader, the President favors business solutions [27]. By extending community care for veterans further and by approving plans for infrastructure assessment, he may be sending a message that the VHA is inefficient.

#### 1.5.4. The Media

The media is likely to report incremental changes to the veteran’s healthcare system which result in negative outcomes are likely to be reported widely. As in the initial scandal, the media can make a difference [28].

### 1.6. The Political Environment

Given the current political environment, additional major changes in veterans’ healthcare may not be supported. Without bi-partisan support for additional interventions, policy changes would require executive orders and the rule-making process. It is here where the courts may have a significant role.

### 1.7. Synopsis

As the VHA continues to struggle with executing its mission, Congress and the President have stepped in with money and programs to attempt to fill the void. Despite the lackluster performance of the Veterans Choice program, there may be reasonable hope that the VA Mission Act will improve veterans care. The question then becomes what other policy interventions are available.

## 2. Materials & Methods

For this policy analysis, the framework of MacRae and Wilde is loosely followed [29]. The use of utility (decision or performance) matrices in decision-making has been promulgated for health interventions [30] and that is the approach used here. A utility matrix evaluates health policy interventions against policy goals. Assume that three policies, {*A*, *B*, *C*}, are being evaluated against three operationalized criteria, {*X*, *Y*, *Z*}. Qualitative or quantitative analysis of each potential policy against each criterion is made based on the definitions provided for the criteria. Once all assessments are completed for all criteria and all policies, the assessments for each criterion across all policies are converted to ranks. For this policy analysis, lower values reflect better rankings. Decision makers often weight criteria based on relative importance or political consequences and these weights are applied to each ranking within that criterion. If the weights across all criterion sum to the number of criterion (e.g., the sum of the weights equals 3 in this case), then the sum of the values in the weighted table will be identical to the sum of values in the unweighted table. Sensitivity analysis is often conducted by manipulating the weights to identify under what weighting conditions other policy interventions might emerge. Table 1 discusses the use of a decision matrix for this example.

### 2.1. Goals and Objectives of Public Policy for the VHA Problem

The U.S. government and public in general expect veterans to receive timely access to quality, cost-effective care. These principles reflect the “Iron Triangle of Healthcare” promulgated by William Kissick and form three of the evaluation criteria: cost, quality and access [31]. Further, any evaluation of policies should consider analysis of political feasibility [32], as well as administrative feasibility (ability to obtain resources and administer the policy). Operational definitions for each selected criterion are then required.

Healthcare costs are difficult to assess even with assembled panels of experts. In the case of this analysis, annual cost per beneficiary is the primary measure when available. If this metric cannot be estimated (as in the case of one policy intervention), then comparative analysis of fixed and variable costs using the status quo is used. Cost is measured in dollars with lower costs indicating a better solution for this criterion.

Measuring quality associated with a policy intervention is difficult, particularly when fraudulent or inaccurate reporting of metrics may exist by all parties involved. For example, a GAO audit recently identified that the VHA does not capture data from all providers including contract providers as well as advanced practice providers, so the reported quality metrics are a non-random subsample. Further, the audit indicated that the VHA does not oversee its medical centers’ monitoring efforts, which is inconsistent with federal controls [33]. Also problematic is that the VHA has been violating federal law for at least 15 years by hiring clinicians whose licenses have been revoked in other states. For example, a neurosurgeon whose license was revoked was hired by the Iowa City VA based on VA’s national guidance [34]. Given the VHA’s recent scandals, confidence in their self-reported quality metrics should be low. In this study, quality will be measured by the patient experience metrics in the most recent Hospital Compare dataset [35]. The Medicare Hospital Compare dataset provides standardized information for all hospital facilities which receive Medicare reimbursement including patient satisfaction scores. Because the VHA quality outcome data are flawed, comparing VHA versus non-VHA facilities must be done on identically gathered and reported reliable data. The data that best meet this criterion are the Hospital Consumer Assessment of Healthcare Providers and Systems (HCAHPS) patient satisfaction data gathered by Hospital Compare and the VHA equivalent, the Survey of Healthcare Experiences of Patients (SHEPS). Both instruments contain 11 identical HCAHPS questions, which may be quantitatively used to assess quality. These surveys provide reasonable comparisons of quality across systems [36]. One should note that some studies have shown that VHA facilities provide at least the same quality as non-VHA facilities using the VHA’s non-random subsample (and flawed reporting), while satisfaction may be lower [37,38].

Access is a difficult variable to define, as most associated metrics fail to consider individuals who wanted access and were not provided it. VHA reports patient access data on its website [39]; however, there is no analogous report for civilian facilities and some of the VHA reporting appears to contain errors. Examples of the problem were seen when evaluating bi-monthly access reports from January through June 2017 (*N* = 11 reports). Of specific interest was the variability of the visit counts and the variability of the proportion of appointments made within 30 days. For facilities with an average appointment count between 10,000 and 12,000 during the survey time (*n* = 30), one facility’s standard deviation for appointment counts was 82.4 over this 6-month period versus 751.9 for all others, a statistical outlier with F_(319,11)_ = 83.2, *p* < 0.001. This same facility’s variation in the proportion of 30-day appointments provided averaged 99.3% (with no reports of 100%) and the standard deviation was 0.0006% compared to 0.1245% for all other facilities (none of which had reports of 100% availability), F_(319,11)_ = 214.9, *p* < 0.001. As another example, the coefficient of variation for appointment counts (CV = s/μ × 100), a measure of standardized variability, was less than one (CV = 0.694) for a facility with on-average 5622 patient appointments per month. That same facility had 3 out of 11 reports with identical proportion of appointments within 30 days (99.37%). While these examples might reflect true outliers, they and other statistics are somewhat suggestive of reporting errors. There is also evidence from late 2017 that VHA access metrics are problematic [11]. Given that access metrics for non-VHA facilities are not available and that VHA access metrics may reflect actual waiting time, access will be measured as the number healthcare venue options veterans are provided. If a veteran has access to only one system, this would be worse than having access to two or more systems. While this is an imperfect measure, it allows for policy comparison at least at the macro level.

Both political and administrative feasibility are assessed qualitatively. Political feasibility is assessed via ranking of policy alternatives based on known or inferred positions of actors, an evaluation of the current environment and an assessment of events that would either favor or not favor a particular policy. Policies assessed to be more politically palatable are deemed better than others. Administrative feasibility is a ranking assessment of ease in which a plan might be implemented based on required resources and overhead. Some of these resources include facilities as well as personnel. Where possible, evidence of administrative complexity from the baseline status quo is provided.

### 2.2. Weighting

Weighting of decision matrices is subjective and should be coupled with sensitivity analysis. To assign weights, Table 2 is used to depict importance values which are converted to weights. The sum of the importance assessment by criterion is {8, 10, 10, 7, 6} for {cost, quality, access, political feasibility, administrative feasibility}, respectively. Converting these to weights (dividing each value by the sum of the criteria importance and multiplying by the number of criteria) results in starting weights of {0.98, 1.22, 1.22, 0.85, 0.73} for each of the respective criteria.

### 2.3. Policy Options

With the operational definitions of the criteria complete, an analysis of reasonable policy options is necessary. Here, we explore four possible policy interventions: expansion of the VHA system to provide all required veteran care, phased elimination of the VHA in favor of central payment system at the Centers for Medicare and Medicaid (CMS), expansion of military retiree subsidized insurance plan to include all veterans regardless of retirement status (private insurance), retention of the status quo. All interventions except for the status quo would require legislative action. Each of these policy options might achieve the goals of providing cost-effective, quality care for veterans.

The first option (short title: VHA only) expands the VHA sufficiently to address the unmet demand of veterans. This would return the treatment currently outsourced to the VHA. To do so would require an understanding of where that demand exists and in what quantity.

The second option (short title: CMS) is a long-term, phased elimination of the VHA in favor of a central payment system. This plan would leverage facility closings or acquisitions to divest VHA assets while simultaneously expanding the VA Mission Act so that all veterans would receive care in private facilities. Funding control and program rules would logically move to the Centers for Medicare and Medicaid (CMS). Entitled veterans would receive this care without additional payment.

A third option (short title: PI) would expand the current, subsidized Department of Defense insurance program (TRICARE Prime managed care plan, TRICARE Select fee for service plan and TRICARE for Life Medicare wraparound coverage) to the entitled veteran population. For TRICARE Prime, veterans would be required annual payments, which are currently a fraction of the actual cost of care. (Annual plan rates for 2018 are $289.08 with no deductible and nominal co-pays.) For TRICARE Select, the veteran would need to cover an annual deductible (currently $150 for an individual), co-pays, an annual enrollment (currently $450 for retirees) and out of network charges if appropriate. Some or all of these costs might be waived. For TRICARE for Life, veterans would only pay the Medicare Part B premiums [40].These insurance plans would need to be negotiated among regional providers, the Department of Defense and the Department of Veterans Affairs. A logical incrementalism approach would eventually merge the VHA functions with the DoD, phasing out VHA’s direct treatment responsibilities.

The status quo option (short title: SQ) retains the current VHA Mission Act components. This Act effectively mixes an NHS-style system (the VHA) with an NIH-component (centralized payment to non-government run facilities). Some of the details of the plan as well as long-term funding have yet to be established; however, the intent is to provide all required care by allowing eligible veterans to receive treatment in the VHA system or in non-VHA facilities under certain circumstances.

## 3. Results

The policies presented each have secondary and tertiary effects. These will be explicated here by evaluating cost, quality, access, political feasibility (including public expectations) and administrative feasibility. For those unfamiliar with decision matrices, a review of Table 1 up-front might be helpful prior to reading the alternative assessments.

### 3.1. Option 1: VHA-Only Assessment

Cost. The VHA-only option would require expansion in facilities and resources to handle unmet demand. If the magnitude of unmet demand is calculated as a proportion of annual community care payments ($14.2 billion) over all annual costs ($98.0 billion), then the unmet demand would be nearly 14% of total workload. However, the $98.0 billion reflects many VHA fixed costs rather than variable costs of care, so the 14% estimate may be low, even if the variable costs of the VHA are cheaper. To accommodate a 14% increase in the VHA system, facility and materiel acquisitions as well as faculty and staff would be required at some level and those costs are dependent on the way the VHA expands [41]. Further, “VA faces challenges regarding the reliability, transparency and consistency of its budget estimates for medical services, as well as weaknesses in tracking obligations for medical services and estimating budgetary needs for future years” [42]. A VHA-only option is assessed to be more expensive than the status quo ($14.0 K/beneficiary) at least in the short term due to the increase in construction with associated fixed costs.

Quality. An analysis of the SHEP items for patient satisfaction (1 April 2016 through 31 March 2017) against the identical HCAHPS items for all other facilities revealed lower satisfaction on 9 of 11 of the identical metrics (Table 2). Under the binomial assumption of equal likelihood, the probability that 9 or more of the 11 identical measures would be higher for “other facilities” is small (0.032); however, the size of the proportional distances is small except for the pain control measure. Quality based on patient experience metrics is assessed to be lower in the VHA.

While hospital outcome analysis is not accurate due to reporting issues in the VHA, a newly-released report suggests that VHA nursing homes performed poorly on quality compared to their community counterparts during the 2017 calendar year. Table 2 illustrates data acquired by USA Today and the Boston Globe [43]. One metric that seems particularly problematic in Table 3 is that 8.51% of VHA residents at high-risk experienced decubitus ulcers during this reporting period. These are “never happen” events. Given these metrics, it would be hard to justify an assessment that the VHA delivers higher quality healthcare than its civilian counterparts.

Access. Veteran access under an expanded VHA system should improve under this proposal. However, a National Public Radio (NPR) study demonstrated the VHA struggled to execute expanded care when provided additional funds [15]. Further, the unmet demand may be more significant than anticipated and adding facilities may just remove backlog associated with on and off-the-books waiting lists. Evidence of this backlog includes a March 2018 report from the VA Inspector General which identified critical deficiencies at the Washington, DC VA Medical Center, including more than 10 thousand open and pending prosthetic and sensory aid consults [44]. Further, availability for care in only one system is defined as reduced access, as veterans’ options are limited.

Political Feasibility. Given the predilection of the current and previous administrations to provide outsourced community care to support the veterans and the multitude of ongoing oversight actions, it is unlikely that a return to a VHA-only system would be palatable at this point. The leadership changes over the past several years suggest a lack of confidence in the VA in general. Since veterans group generally support the VHA [25], this option is likely to achieve significant levels of public support.

Administrative Feasibility. Administering a VHA-only policy would certainly be more problematic and likely more expensive than the status quo solution. This policy would be a multi-year transition during construction and hiring and during that time, access would continue to be a problem without the continued use of community care options. While the VA Mission Act would provide the community care option, the VHA would have to implement real systems capable of supporting that access during the transition. The VHA’s history in supporting payments to third parties under legislative directive is suspect as evidence by its failures under the Veterans Choice Act [45].

### 3.2. Option 2: CMS Assessment

Cost. For this policy option, CMS gradually takes over funding of all veteran healthcare through its existing Medicare processes. The fixed costs associated with the VHA-operated facilities (roughly $6.2 billion in FY2019) would phase out over time and facilities as well as equipment would be divested. While the fixed costs of care would disappear, the variable costs would likely but not necessarily rise. As indicated by the CBO, the VHA’s actual knowledge of current costs is poor [41]. While dated studies relying on data from 1999 or earlier indicated that the VHA care was less expensive than private-sector care, the cost comparisons may not be relevant now due to changes in patient mix, demand and so forth. Further, the cost-effectiveness of that care is unknown and “comparing health care costs in the VHA system and the private sector is difficult partly because the Department of Veterans Affairs [6], which runs VHA, has provided limited data to the Congress and the public about its costs and operational performance” [41]. The most recent study (VA sponsored and dated) from Nugent et al. in 2004 evaluated six non-randomly selected facilities to estimate cost-savings over Medicare between 17–20% [46]. That study is not relevant today. First, 49% of the estimated “cost offsets” based on an inflated workload estimate were associated with the VA’s ability to negotiate pharmaceutical prices ($81, 946/$167, 383). Fourteen years ago, that estimate might have been reasonable. With Medicare Part D, CMS has demonstrated the bargaining power to reduce pharmaceutical costs by 55% [47]. Second, adjustments of 3.3% for corporate overhead, interest on capital assets and torts were included in the authors’ study; however, legal settlements in the VA more than tripled from 2011 to 2015 going from $98 million to $338 million [48].

Aside from the Nugent’s study cost analysis, one can compare the estimate of $14.0 K per enrolled veteran per year with the Medicare spending provided by the Henry J. Kaiser Foundation and derived from CMS and Census Bureau data. The per enrollee Medicare spending in 2014 was $10,986 [49]. Inflating these values using the Bureau of Labor Statistics inflation rates from May 2015 through May 2018, {2.8%, 3.1%, 2.7%, 2.4%} [50], results in a revised estimate of $12.2 K per beneficiary. This would imply that Medicare expenditures per patient with access may be less than the VHA.

Quality. CMS relies on Medicare approved facilities for providing care. As national insurance for the elderly, it demands and incentivizes quality through a variety of mechanisms such as Accountable Care Organizations (ACOs), accreditation and Quality Improvement Organizations. Thus, shifting care from VHA facilities to private facilities which accept Medicare should result in quality at least as good as the VHA. From a patient experience view, non-VHA facilities scored better than VHA facilities on 9 of 11 common satisfaction survey items as discussed earlier. Table 2 contains the DoD satisfaction results. A Friedman’s non-parametric repeated measures test for satisfaction scores indicated differences among the three groups ($\chi_{2}^{2}=16.545,\;p=0.0002$). Wilcoxon Rank Sum post-hoc tests found differences between non-VHA and VHA (*V* = 57.5, *p* = 0.033), between VHA and DoD (*V* = 66, *p* = 0.004) and between DoD and non-VHA (*V* = 64, *p* = 0.003). All tests were performed in R Statistical Software [51].

Access. With a logical incrementalism approach and the opportunity for large systems to acquire VHA facilities built into this option, access to care should be better than the VHA only system, as veterans would have access to all Medicare-accepting organizations. This option expands choice and would therefore be preferred over the VHA-only option.

Political Feasibility. Members on both sides of the aisle as well as some veterans’ groups (i.e., the Veterans of Foreign Wars) have indicated they are not in favor of VHA “privatization” [25]. The word “privatization” is politically charged; therefore, many who actually are in favor moving towards a single payer system avoid it [25]. Still, open discussion of a CMS option would be politically charged and the public would likely take their cues from both the veterans groups and media. Implementation of a plan such as this would require strong leadership and face many challenges. Arguably, the simplest method to implement this plan would be incrementally without discussion.

Administrative Feasibility. While the CMS already manages Medicare, the inclusion of payments on behalf of veterans would be a new mission and difficult to execute. Further, the sale and extraction of residual from the existing VHA properties would require significant planning. Administratively, this plan would likely be more difficult to implement than the VHA only option.

### 3.3. Option 3: PI

Cost. Option 3 expands TRICARE subsidized insurance to veterans. In 2012, the Department of Defense spent $52 billion on TRICARE to cover 9.5 million beneficiaries, an average of $5.5 K per covered individual [46]. Inflating this to 2018 dollar using the BLS medical inflation rates for May 2013 through May 2018 [52] results in a revised estimate of $6.5 K per beneficiary. While some might suggest that the lower cost per beneficiary user is due to case mix, this may or may not be true. Post-traumatic stress disorder, polytrauma combat veterans, burn victims and so forth are part of the DoD as well as the VA. Further, dual eligible veterans (those who are entitled to care from the VHA) also seek care from TRICARE. The cost structure of TRICARE appears to be better than either a VHA only option or the CMS option.

Quality. TRICARE is an insurance-based program. Outcomes are likely to be similar to the other two options. When comparing the most recent patient experience metrics for the DoD (a proxy for TRICARE) [53] versus all other facilities and the VHA using the Hospital Compare data, the DoD metrics are superior to the VHA for all 11 questions and are superior in 10 of 11 categories to all other facilities. In both cases, the results are unlikely due to chance alone (*p* < 0.001, *p* < 0.006 respectively using binomial models). In terms of patient experiences, this option is preferred over VHA-only and CMS.

Access. TRICARE Prime provides access through participating providers both within and outside of the military healthcare system. Because TRICARE includes both a managed care and indemnity insurance option, the access to care is better than a VHA-only system and similar to that of the status quo. The CMS option arguably provides more options for access, as the acceptance rate for new patients in 2015 was 72% for Medicare versus 67% for TRICARE in 2012 [54].

Political Feasibility. While the consolidation of veterans’ care with DoD beneficiary care may make sense from the aspects of cost, quality and access, the political feasibility would be problematic. Transitioning to DoD insurance plans might (1) be a non-clandestine threat to the existence of the VHA; (2) require contributions from veterans unless waived, which would be highly unpopular and (3) be opposed by many in Congress whose constituencies include many veterans. From a feasibility perspective, this option probably ranks below VHA-only and incremental transition to the CMS.

Administrative Feasibility. From an administrative viewpoint, this option is feasible. The VHA already uses one of the major TRICARE contractors (Health Net Federal Services) to support the existing Veterans Choice program [55,56]. Combining TRICARE and (in the future) VA Mission Act contracts would be feasible, arguably more feasible than expanding the VHA-only option.

### 3.4. Option 4: SQ

Cost. The status quo option costs are currently estimated to be $14.0 K per treated veteran per year. Based on the previous cost analyses, this option is probably only superior to the VHA-only option and inferior to all others.

Quality. Under the status quo, outcome quality should be at least as good as all other plans; however, experience of care (satisfaction) metrics should be a mix of the VHA current scores and the other facility scores in Table 3. Using this logic, the status quo is superior to VHA-only but inferior to all other options on this criterion.

Access. With access to the VHA and contracted facilities, veterans should have more choice than the VHA only option but less choice than PI or CMS. PI and CMS provide a wider array of options.

Political Feasibility. Since this is the status quo solution, it has proven to be palatable. In fact, the expansion of community care indicates that support for expanding such action exists. This intervention ranks first in political feasibility.

Administrative Feasibility. This criterion might be an issue despite the VA Mission Act having been enacted already. The responsibility for coordinating the outside care for veterans lies within the VHA. The question remains whether the VHA be able to improve upon its demonstrated performance with the Veterans Choice Act. Still, there is some experience in contracting care within the VHA, so it probably ranks first in administrative feasibility.

### 3.5. Comparisons

The use of the evaluation criteria provides a mechanism for building a combined evaluation and comparison matrix, both weighted and unweighted (Table 4). The unweighted and weighted solutions both favor PI, the TRICARE insurance option. This option was ranked first or second in all but one category, political feasibility. In the unweighted matrix, both CMS and SQ solutions tied for second, while CMS took second position by itself under weighting. The VHA only solution was assessed to be the worst solution weighted and unweighted, primarily because of quality metrics and access metrics.

For the CMS policy intervention to be selected as the weighted choice, the weight for access would have to increase to nearly 2.0 with all other criterion being held constant. Alternatively, the weight for political feasibility would have to increase above 1.22. For SQ to be selected, the weight for either political feasibility or for administrative feasibility would need to be increased above 2.4 with all other weights held constant. VHA-only is a dominated solution that is never selected under any weighting scheme. Given this analysis, the PI or CMS solutions are better options than the status quo or VHA only; however, the PI is preferred due to the better cost and quality metrics.

## 4. Discussion

The United States has a long-standing obligation to provide veterans access to high quality care. Failures in the system have resulted in unnecessary morbidity and mortality. The country has an obligation to provide a lasting solution to wicked problem that balances the components of the “Iron Triangle”.

The policy analysis suggests that PI is probably the best solution and it is certainly worth exploring. This solution, however, might not be politically viable. The CMS solution, on the other hand, may be implemented through incremental steps such as the expansion of the VA Mission Act and appears to be reasonably implementable. The current status quo provides an access relief valve but fewer choices and additional costs, particularly when compared to the PI solution; however, expanding the VA Mission Act incrementally might achieve a permanent solution to the problem.

Any policy analysis always has major limitations. While care is taken in the selection of criteria, there is no perfect method for doing so. An infinite number of policy interventions are available and subsets of these might be evaluated differently. Weighting selection for criteria are subject to debate. Analysis of rankings on selected criteria may be second-guessed. But the objective analysis of potential policies serves one purpose well: it invokes debate.

The VHA has been involved in repeated scandals since 2014 and yet it is the veterans who suffer. Leadership at all levels of the Government have an obligation to fix these problems. The VA Mission Act passed by the Congress and signed by President Trump may be a good start but it relies on the VHA for implementation. Should the public place its confidence yet again in this organization? More importantly, should our veterans?

## Figures and Tables

**Table 1:** This table illustrates a typical weighted utility matrix. The left-hand column contains the policies under consideration, {*A*, *B*, *C*}. The top row contains the criteria for evaluating the policies, {*X*, *Y*, *Z*}. The assessed values for each criterion across all policies sum to 6, 1 + 2 + 3. Ties receive an average of the occupied position as illustrated under criterion *Z* where 2 values of 2.5 exist representing a tie for positions 2 and 3 for policies *A* and *C*, (2 + 3)/2 = 2.5. Since lower values indicate a higher assessment, the unweighted decision would be policy A, as its sum is 5.5 compared to 6.0 and 6.5. Weights are assigned to each criterion such that the sum of those weights (0.5 + 0.5 + 2.0) equals the number of criteria (3) to keep the magnitude of the unweighted and weighted decisions the same. (Both the unweighted and weighted solution values sum to 18). The weighted decision values are calculated by applying the weights to the assessed values for each policy. For example, policy *A*’s weighted solution is calculated as follows: 0.5 × 1.0 + 0.5 × 2.0 + 2.0 × 2.5 = 6.5. In this example, the weighted decision would be policy *B* versus policy *A*. The weights are often adjusted to provide sensitivity analysis.

	Criteria			Decision	
Policy	*X*	*Y*	*Z*	Unweighted	Weighted
*A*	1.0	2.0	2.5	5.5	6.5
*B*	2.0	3.0	1.0	6.0	4.5
*C*	3.0	1.0	2.5	6.5	7.0
Sum	6.0	6.0	6.0	18.0	18.0
Weights	0.5	0.5	2.0	Lower is better.	

**Table 2:** Patient satisfaction scores for 1 April 2016 through 31 March 2017 for the VHA, non-VHA and DoD.

Item: Patients Who	VHA	Other Facilities	DoD
1. “Strongly Agree” they understood their care when they left the hospital	0.554	0.521	0.605
2. gave their hospital a rating of 9 or 10 on a scale from 0 (lowest) to 10 (highest)	0.684	0.727	0.716
3. reported that staff “Always” explained about medicines before giving it to them	0.663	0.651	0.752
4. reported that the area around their room was “Always” quiet at night	0.566	0.622	0.681
5. reported that their doctors “Always” communicated well	0.779	0.818	0.868
6. reported that their nurses “Always” communicated well	0.763	0.802	0.857
7. reported that their pain was “Always” well controlled	0.640	0.710	0.735
8. reported that their room and bathroom were “Always” clean	0.733	0.742	0.766
9. reported that they “Always” received help as soon as they wanted	0.649	0.682	0.785
10. reported that YES, they were given information about what to do during their recovery at home	0.871	0.873	0.907
11. reported YES, they would definitely recommend the hospital	0.695	0.720	0.727

**Table 3:** The 2017 quality care comparison for nursing homes does not reflect case complexity; however, certain measures such as the administration of anti-psychotics, catheter mistakes and decubitus ulcers are problematic.

Measure	VHA	Non-VHA
Reported Pain within the Past Five Days	32.64%	5.59%
Received Anti-Psychotic Medication, which the FDA has Associated with an Increased Risk of Deaths in Elderly Dementia Patients	20.89%	15.48%
Experienced Marked Decrease in Abilities to Perform Acts of Daily Living	16.70%	14.99%
Had a Catheter Left in Their Bladder, which can lead to Urinary or Blood Infections and Other Complications	11.96%	1.83%
High-risk Residents with Serious Bed Sores, which may be Prevented by Repositioning/Cushioning	8.51%	5.57%

**Table 4:** Evaluation and comparison of possible policy interventions is depicted. The unweighted and weighted recommendations are initially identical without sensitivity analysis and in favor of the TRICARE option.

	Criteria					Decision	
Policy	Cost	Quality	Access	Political	Admin.	Unweighted	Weighted
VHA Only	4.0	4.0	4.0	3.0	3.0	18.0	18.4
CMS	2.0	2.0	1.0	2.0	4.0	11.0	10.2
PI	1.0	1.0	2.0	4.0	2.0	10.0	9.5
SQ	3.0	3.0	3.0	1.0	1.0	11.0	11.8
Sum	10.0	10.0	10.0	10.0	10.0	50.0	50.0
Weights	0.98	1.22	1.22	0.85	0.73	Lower is better.	
